# Cardiac Angiosarcoma-Associated Membranoproliferative Glomerulonephropathy

**DOI:** 10.1155/2011/956089

**Published:** 2011-05-18

**Authors:** Lokesh Shahani, Michael Beckmann, Srikanth Vallurupalli

**Affiliations:** ^1^Department of Internal Medicine, Southern Illinois University School of Medicine, 701 N. First Street, Springfield, IL 62702, USA; ^2^Pathology Associates of Central Illinois LTD, Springfield, IL 62702, USA

## Abstract

Primary cardiac angiosarcoma is a rare cardiac tumor. The initial clinical course is often asymptomatic, and metastatic disease is present in a majority of affected patients at diagnosis. We present a patient who presented with a hemorrhagic pericardial effusion. No malignant cells were evident on cytological examination. He subsequently developed membranoproliferative glomerulonephritis requiring hemodialysis. Metastatic cardiac angiosarcoma was diagnosed 5 months later. To our knowledge, this is the first reported case of paraneoplastic membranoproliferative glomerulopathy associated with cardiac angiosarcoma.

## 1. Introduction

The occurrence of paraneoplastic glomerulopathies in patients with cancer is well recognized [1]. We report a patient with cardiac angiosarcoma who initially presented with a pericardial effusion and paraneoplastic glomerulopathy.

## 2. Case Report

A 79-year-old Caucasian male presented with gradually worsening shortness of breath and fatigue. A chest X-ray revealed pulmonary congestion and cardiomegaly. Lab tests revealed normal kidney function. A transthoracic echocardiogram revealed a large pericardial effusion with features of cardiac tamponade. Hemorrhagic fluid was obtained at pericardiocentesis, and around 400 milliliters were drained. Cytology was negative for malignant cells. Bacterial cultures grew *Staphylococcus epidermidis*. Though the clinical picture was not suggestive of bacterial pericarditis, he was treated with intravenous vancomycin for 4 weeks.

**Figure 1:**
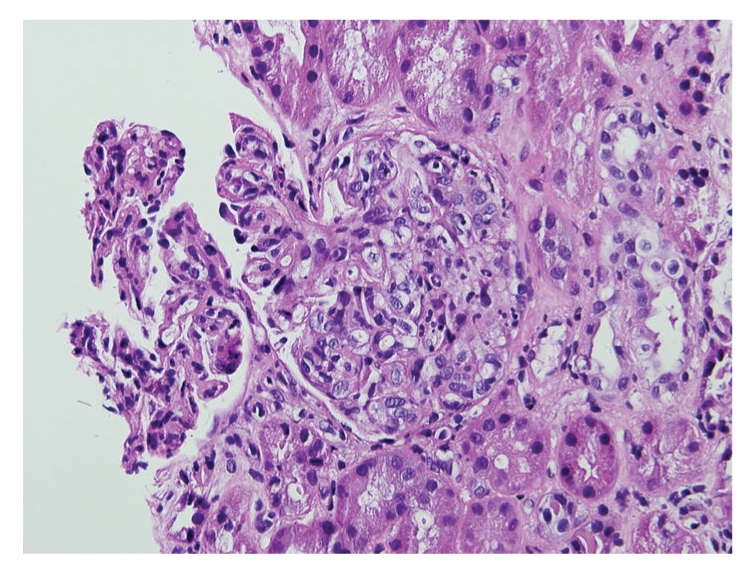
H & E stain of renal biopsy: Light micrograph suggestive of membranoproliferative glomerulonephritis showing hypercellular glomeruli with prominence of mesangial matrix.

**Figure 2:**
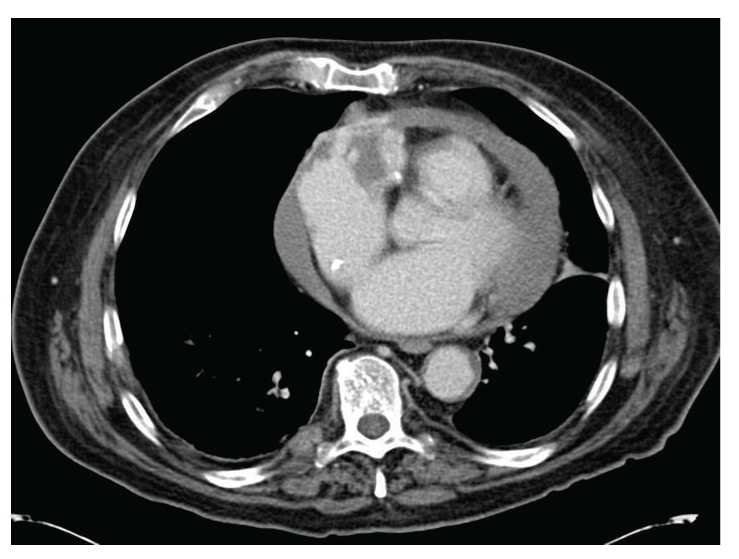
Computed tomography scan of the chest showing the right atrial mass and pericardial effusion.

**Figure 3:**
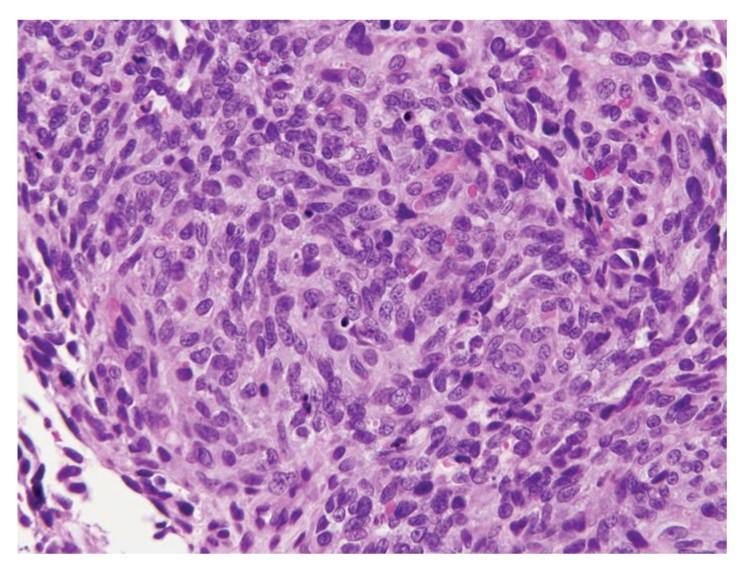
H & E stain of the liver biopsy—angiosarcoma with hyperchromatic spindle cells.

A month later the patient developed shortness of breath and progressive pedal edema. Laboratory studies revealed a serum creatinine of 8.8 mg/dL. Spot urine protein to creatinine ratio was 3.1. He was found to have low complement C3 and normal C4 levels. Serum ANA, ANCA, hepatitis serology, and serum and urine protein electrophoresis were negative. A repeat transthoracic echocardiogram revealed a small pericardial effusion and normal cardiac function. A renal biopsy was performed. Light microscopy revealed hypercellular glomeruli with some prominence of mesangial matrix (Figure 1). Immunofluorescence staining demonstrated granular glomerular basement membrane and variable mesangial staining with IgG, IgM, C3, C1q, kappa, and lambda. Tubular basement membranes and arterioles stained positive with C3. Electron microscopy demonstrated scattered small electron dense immune deposits within the mesangium and located primarily below the glomerular basement membrane reflection. There were no features suggestive of postinfectious glomerulonephritis or vancomycin-induced renal injury. Though C1q staining is not typical, the combined light, immunofluorescence, and electron microscopic findings were felt to be consistent with a diagnosis of type 1 membranoproliferative glomerulonephritis. The patient was started on thrice weekly hemodialysis. Five months later, the patient developed gradually worsening diffuse abdominal and lower back pain. An abdominal and pelvis CT noted multiple hepatic and spinal lesions suspicious for metastasis. In addition, a heterogeneously enhancing mass with features of central necrosis was present in the right atrioventricular groove invading the wall of the right atrium (Figure 2). An ultrasound-guided needle core biopsy of a hepatic lesion revealed an angiosarcoma (Figure 3). This was presumed to be of cardiac origin based on the imaging features. Patient opted for palliative treatment with hospice care. 

## 3. Discussion

Primary tumors of the heart are rare with the incidence varying from 0.001% to 0.03% [2]. Further malignant tumors account for just 25% of the primary cardiac tumors [3]. Angiosarcomas account for around 33% of all primary malignant cardiac tumors. They are however exceedingly rare with an incidence of only 0.0001% in autopsy series [4].

Primary cardiac angiosarcomas rarely cause symptoms until late in the clinical course. Most of the symptoms are due to either pericardial effusion with or without cardiac tamponade, venal caval obstruction, arrhythmias, or related to metastases to other organs [5]. In fact, 66% to 89% of patients have metastatic disease at presentation [6]. Common sites of metastases include the pericardium, lungs, liver, mediastinal lymph nodes, and bone [5].

Paraneoplastic glomerulopathies are rare manifestations of neoplastic disease. Nephrotic syndrome is the most common presentation of these paraneoplastic glomerulopathies. Different glomerular diseases are associated with different neoplasia, such as membranous nephropathy in patients with solid tumors and minimal changes disease which is strongly associated with Hodgkin's lymphoma [7]. Sarcomas have rarely been associated with paraneoplastic glomerulopathy. In a recent review of the literature by Bacchetta et al. [1], 6 cases of sarcoma with paraneoplastic glomerulonephritis were identified [1]. Pathology in three of them was consistent with membranous nephropathy where as there was one case each associated with minimal change disease, focal and segmental glomerulosclerosis, and with mesangiocapillary glomerulonephritis. To our knowledge, no cases of glomerulopathy associated with cardiac angiosarcoma have been reported in the literature.

 A temporal relationship between the glomerulopathy and the cancer is suspected when kidney dysfunction develops 6 months before or after the diagnosis of malignancy [8]. The syndrome often precedes discovery of tumor by several months. This is illustrated by a study which showed proteinuria preceded or occurred at the same time as cancer in 80% of patients [9]. Although the pathological mechanism for paraneoplastic glomerulopathy remains unknown, tumor-associated immune complexes and deposit nephritogenicity have been implicated in glomerular injury and overt neoplastic renal disease [10]. 

In our patient, membranoproliferative glomerulonephritis developed almost five months before the diagnosis of metastatic angiosarcoma. The initial differential diagnosis of the glomerulopathy included vancomycin-induced or postinfectious etiologies. There were, however, no features of drug toxicity on kidney biopsy and no evidence of supratherapeutic levels during drug monitoring. There was no convincing evidence of a postinfectious etiology either. Though the patient was treated as presumptive bacterial pericarditis, the clinical presentation was not consistent with the diagnosis. The coagulase negative staphylococcus may have been a contaminant and was not isolated in any other body fluid. In addition, bacteria rarely cause hemorrhagic pericardial effusion; iatrogenesis and malignancy are much more common causes in the Western hemisphere [11]. *Staphylococcus epidermidis* infection of ventriculoatrial shunts can cause membranoproliferative glomerulonephritis (shunt nephritis). Similar renal effects have been noted in patients with infected central venous catheters [12]. Nephritis typically manifests prior to the start of antimicrobial therapy. The lack of a shunt or indwelling catheter in our patient and the appearance of kidney disease a month after effective treatment of presumed infection make this diagnosis less likely. Investigations for all other causes of MPGN were negative, and the patient was diagnosed to have idiopathic MPGN. 

Cytology of the hemorrhagic pericardial fluid at initial presentation was negative for malignant cells. This is not unusual in the case of cardiac angiosarcoma [13, 14]. The initial echocardiogram did not reveal any mass in the right atrium. The tumor was probably too small to be detected using transthoracic echocardiography, and similar instances have been described in the literature [14, 15]. Though this approach has not been validated in clinical studies, it may be reasonable to obtain magnetic resonance imaging of the heart and pericardium in cases of hemorrhagic pericardial effusion with no obvious pathology. After pericardiocentesis, the patient did not develop recurrence of significant pericardial effusion as one would expect in the case of an untreated malignancy. A similar phenomenon has been described in at least two previously reported cases of angiosarcoma [14, 16]. Though angiosarcoma was diagnosed 5 months after the detection of glomerulonephritis, it was probably already present at the onset of kidney disease and was thus considered the most likely etiology.

## 4. Conclusion

Our case report represents a patient with paraneoplastic membranoproliferative glomerulonephritis as an early presenting feature of cardiac angiosarcoma. It also illustrates the difficulties faced by clinicians in diagnosing primary cardiac angiosarcoma. Pericardial cytology may be negative, and the pericardial effusion may not recur after the initial episode. Transthoracic echocardiography may not be sensitive enough to diagnose these tumors at presentation. Finally, malignancy should be ruled out before classifying glomerulonephritis as idiopathic.
